# A 10-Year Analysis of the Effects of Media Coverage of Regulatory Warnings on Antidepressant Use in The Netherlands and UK

**DOI:** 10.1371/journal.pone.0045515

**Published:** 2012-09-20

**Authors:** Juan Francisco Hernandez, Aukje K. Mantel-Teeuwisse, Ghislaine J. M. W. van Thiel, Svetlana V. Belitser, Jan Warmerdam, Vincent de Valk, Jan A. M. Raaijmakers, Toine Pieters

**Affiliations:** 1 Department of Pharmacoepidemiology and Clinical Pharmacology, Utrecht Institute for Pharmaceutical Sciences (UIPS), Utrecht University, Utrecht, The Netherlands; 2 Julius Centre for Health Sciences and Primary Care, University Medical Centre Utrecht, Utrecht, The Netherlands; 3 IMS Health, Capelle aan den IJssel, The Netherlands; 4 Genees-en hulpmiddelen Informatie Project (GIP – Drug Information Project), Healthcare Insurance Board (CVZ), Diemen, The Netherlands; 5 GlaxoSmithKline, External Scientific Collaborations Europe, Zeist, The Netherlands; 6 EMGO, VU Medical Centre, Amsterdam, The Netherlands; University of Manitoba, Canada

## Abstract

**Background:**

In 2003–2004 and 2007–2008, the regulatory banning of SSRI use in pediatrics and young adults due to concerns regarding suicidality risk coincided with negative media coverage. SSRI use trends were analyzed from 2000–2010 in the Netherlands (NL) and the UK, and whether trend changes might be associated with media coverage of regulatory warnings.

**Methods:**

Monthly SSRIs sales were presented as DDDs/1000 inhabitants/day. SSRI-use trends were studied using time-series segmented regression analyses. Timing of trend changes was compared with two periods of media coverage of warnings. Annual Dutch SSRI prescription data were analyzed by age group.

**Results:**

Trend changes in SSRI use largely corroborated with the periods of media coverage of warnings. British SSRI use declined from 3.9 to 0.7 DDDs/month (95%CI 3.3;4.5 & 0.5;0.9, respectively) before the first warning period (2003–2004). A small decrease of −0.6 DDDs/month (−1.2; −0.05) was observed in Dutch SSRI use shortly after 2003–2004. From 2007–2008, British SSRI use stabilized, whilst Dutch SSRI use diminished to −0.04 DDDs/month (−0.4;0.3). Stratified analyses showed a rapid decrease of −1.2 DDDs/month (−2.1; −1.7) in UK paroxetine use before 2003–2004, but only a minimal change in Dutch paroxetine use (−0.3 DDDs/month −0.8;0.2). Other SSRI use, especially (es)citalopram, increased during 2003–2004 in both countries. Significant reductions in Dutch paroxetine use were observed in pediatrics, adolescents, and young adults after 2003–2004.

**Conclusion:**

Changes in SSRI use (NL & UK) were associated with the timing of the combined effect of media coverage and regulatory warnings. Our long-term assessment illustrates that changes in SSRI use were temporal, drug-specific and more pronounced in pediatrics and young adults. The twofold increase in SSRI use over one decade indicates that regulatory warnings and media coverage may come and go, but they do not have a significant impact on the overall upward trend of SSRI use as a class in both countries.

## Introduction

Health care providers and consumers alike seek health and medical information from the news media and act accordingly, changing their perceptions and behavior [1], [2]. Several studies have documented the effects of media and regulatory interventions on medical decisions, health services utilization, and pharmaceutical sales patterns [3], [4]. The influence of news media reports or pharmaceutical regulatory warnings for antidepressants has been studied. For instance, Martin et al. identified a correlation between increased negative media attention on the safety of paroxetine (a Selective Serotonin Reuptake Inhibitor-SSRI) and the temporal and voluntary reporting of adverse drug reactions (ADRs). The measured decrease in paroxetine prescriptions in England, after 2002, was attributed to regulatory warnings and lawsuits (see Box S1), rather than media reports [5]. Another study also found a temporal decline in pediatric antidepressant prescriptions in the United Kingdom (UK) related to regulatory actions after 2003 [6]. This regulatory-driven fall in antidepressant use in pediatrics was also reported in a study establishing a greater impact of warnings in the UK than in the United States (US) or the Netherlands (NL) from 2003–2005 [7]. Volkers et al. added more evidence to this drop in antidepressant prescriptions (2001–2005) in Dutch pediatric patients [8]; and two other studies also showed the influence of the warnings in the US [9], . However, none of abovementioned studies analyzed the long-term influence of regulatory warnings on antidepressant use; thus, a second set of warnings (updates 2007–2008) were not included in those analyses. In addition, the influence of both warning periods has not been studied in combination with the long-term influence of media coverage, nor has the differential impact on use in various age groups been examined.

In a previous study, we analyzed the long-term dynamics of ‘good’ and ‘bad’ news in scientific journals and Dutch and British newspapers in the context of the SSRIs and suicidality controversy [21]. We found an increase in the number of articles discussing the positive (protective) effect of antidepressants for the treatment of depression or to prevent suicidality in scientific journals. This “positive publication tendency” did not influence the dissemination of negative news in Dutch and British dailies. However, negative reporting in the same newspapers was predominantly about the pediatric use of SSRIs and correlated with regulatory warnings in 2003–2004 and in 2007–2008. We hypothesize that in both the NL and the UK, the use of SSRIs was influenced by the synergetic interaction of regulatory warnings (black box warning and updates) and scientific and media attention to the SSRI and suicidality controversy in 2003–2004 and 2007–2008. The aim of this study was to specifically analyze trends of SSRI use between January 2000 and January 2010 in the NL and the UK. In addition, we evaluated whether trend changes could be associated with the combined and long-term effects of the periods of intense media coverage of the warnings. In the NL, we also analyzed the differential impact of media coverage by the type of prescriber and age group.

## Methods

### Data Source

IMS Health provided monthly antidepressant sales data in the NL and the UK for time trends assessment on a national (aggregated) level. Antidepressant sales data in the NL were available from January 2000 to January 2006 for tricyclic antidepressants (TCAs) and other antidepressants (monoamine oxidase inhibitors (MAOIs), as well as serotonin-norepinephrine reuptake inhibitors (SNRIs, etc.). Sales data for SSRIs were available from January 2000 to July 2010. Antidepressant sales data in the UK were available from January 2000 to January 2010 for all antidepressants. Escitalopram entered the market in August 2004 in the NL and in June 2002 in the UK. The sales data provided by IMS Health consisted of wholesaler information from ambulatory care and hospitals that cover, on average, 90% of the total therapeutic drug sales in the NL and UK. IMS Health also provided monthly Dutch SSRIs prescription data stratified by specialty from January 2000 to January 2010. This dataset was used to ascertain changes in the prescribing habits of general practitioners (GPs), and specialists (psychiatrists, cardiologists, oncologists, etc.). The GIP-database (Dutch insurance data retrieved from ambulatory care; not hospitals) provided yearly aggregate SSRI prescription data stratified by age groups from 2000 to 2010. The GIP-database covers, on average, 83% of the insured population in the NL [22].

### Data Presentation

Sales data were classified into three main groups: a) SSRIs, b) TCAs, and c) other antidepressants (other ADs). IMS Health’s sales data were delivered in standard counts, which is the volume unit used to describe sales per counting unit (i.e., tablet, capsule, etc.), together with the given concentration of the active compound. For each antidepressant, monthly use was converted into defined daily doses (DDD)/1000 inhabitants/day, using the standard counts sold, dosage strength, and monthly population estimates per country. The DDD is the international unit of drug utilization approved by WHO for drug utilization studies and is defined as the average maintenance dose of the studied drug when used for its major indication in adults [23]. Yearly Dutch SSRI use in DDD/1000/day per age groups (GIP-database) was adjusted for the age distribution of the population. Monthly Dutch population estimates, as well as yearly age-group population estimates (per strata), were obtained from the Office of Statistics Netherlands (CBS), and UK estimates from the European Commission statistics database (Eurostat) [24], [25].

### Age Groups Categorization (NL only)

The age groups were defined as pediatrics (0–14 years old), adolescents (15–19 years old), young adults (20–24 years old), adults (25–64 years old), and elderly (65 years and older). However, the GIP data combined the use of antidepressants for 15 to 24-year-olds between 2000–2001 hindering a differentiation between adolescents and young adults. Therefore, the ratio of use for adolescents and young adults in 2002–2010 was used to extrapolate use in 2000–2001.

### Periods of Intense Media Coverage of Regulatory Warnings

Based on our analysis of scientific and newspaper coverage, we chose the following periods of intense media coverage of regulatory warnings: a) January 2003 to December 2004, and b) January 2007 to December 2008. The control periods were: a) January 2000 to December 2002, b) January 2005 to December 2006, and c) January 2009 to December 2009 [21].

### Statistical Analyses

To assess whether trend changes in antidepressant use were associated with the combined and long-term effects of both periods of regulatory warnings and scientific and newspaper coverage, we performed time-series analyses for overall SSRI, TCA and other ADs use, and per specific SSRI. The algorithm that describes the principle of our time-series analyses based on change-points was previously reported [26], [27]. This algorithm creates segments within the time-series under two distinct circumstances. First, each segment is created based on the change of the slope over time by fitting linear regressions with autoregressive (AR) models of the second order for random error to correct for the autocorrelation of monthly medication use over time. Second, if the average change of the slope is similar, but there is excessive variation, then a segment is created. The predicted values at the end of a segment and at the beginning of the consecutive segment were fitted as closely as possible. The segment with the lowest minimal number of change-points was selected. Segments were created without consideration of the periods of media coverage of regulatory warnings; however, the selected segments were compared to determine if they coincided with these periods.

Differences in SSRI use (mean) within Dutch age groups were compared with an ANOVA test, assuming that the means of each age group were equal. A Tukey HSD (honest significant difference) post-hoc test was used to determine which age group’s means were significantly different from one another. Statistical significance was set at P<0.05. Analyses were performed using the statistics software program “R” version 2.12.2 [28].

## Results

The use of SSRIs increased in the NL from 16.7 in January 2000 to 27.9 DDDs/1000/day in July 2010, while in the UK, SSRI use doubled from 24.7 in January 2000 to 50.1 DDDs/1000/day in December 2009. The use of other ADs increased from 3.3 in 2000 to 8.3 DDDs/1000/day in December 2005 in the NL, and from 3.4 in 2000 to 12.1 DDDs/1000/day in December 2009 in the UK. TCAs use increased from 4.2 in January 2000 to 5.2 DDDs/1000/day in December 2005 in the NL, whereas in the UK, TCAs use increased from 9.5 in January 2000 to 10.6 DDDs/1000/day in December 2009. On average, the UK population used 1.5-fold more SSRIs, 1.1-fold more other ADs, and 2.1-fold more TCAs than the Dutch did; both populations are comparable with respect to gender and age distributions (Table 1).

**Table 1:** Demographics for the Netherlands and the United Kingdom (2000–2009).

	Netherlands					United Kingdom				
Population characteristics	2000		2009		Growthrate (%)	2000		2009		Growthrate (%)
Population	15,987,075		16,574,989		**3.7**	58,981,904		61,990,973		**5.1**
**Gender**										
Female (%)	8,017,633	(50.5)	8,329,391	(50.5)	**3.9**	30,296,500	(50.7)	31,399,890	(50.6)	**3.6**
**Age groups**										
0–20 Y	3,873,008	(24.4)	3,933,585	(23.9)	**1.6**	12,076,300	(20.2)	11,227,401	(18.1)	−**7.0**
20–65 Y	9,838,500	(62.0)	10,080,387	(61.1)	**2.5**	38,362,500	(64.2)	40,680,109	(65.6)	**6.0**
>65 Y	2,152,442	(13.6)	2,471,815	(14.9)	**14.8**	9,316,600	(15.6)	10,083,462	(16.3)	**8.2**

### SSRI use in the NL and the UK

**Figure 1:**
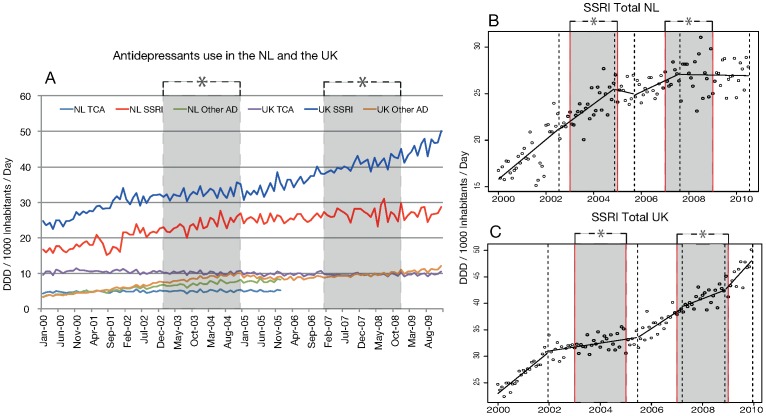
Antidepressant use in the NL and the UK (SSRIs, TCAs, and other antidepressants) (A), segmentation of SSRI use in the NL (B), and in the UK (C). Dotted lines represent a change in use trend and therefore a new, or the end of a segment. *The grey periods represent the periods of media coverage of regulatory warnings.

Regression analyses indicated a short and temporal effect of the regulatory warnings on overall SSRI use in the NL. From 2000, SSRI use increased in a trend that continued until November 2004 (Figure 1A+B, appendix table). After the first period of intense media coverage of regulatory warnings, the growth trend slowed until September 2005 when it increased again until August 2007. SSRI use then plateaued, after the second period of intense media coverage of the warnings and stagnated until July 2010. SSRI use in the UK showed no negative trends during this period, with episodes of rapid increase outside the periods of media coverage of regulatory warnings and episodes of slowed growth during the periods of media coverage of regulatory warnings (Figure 1A+C, appendix table).

**Figure 2:**
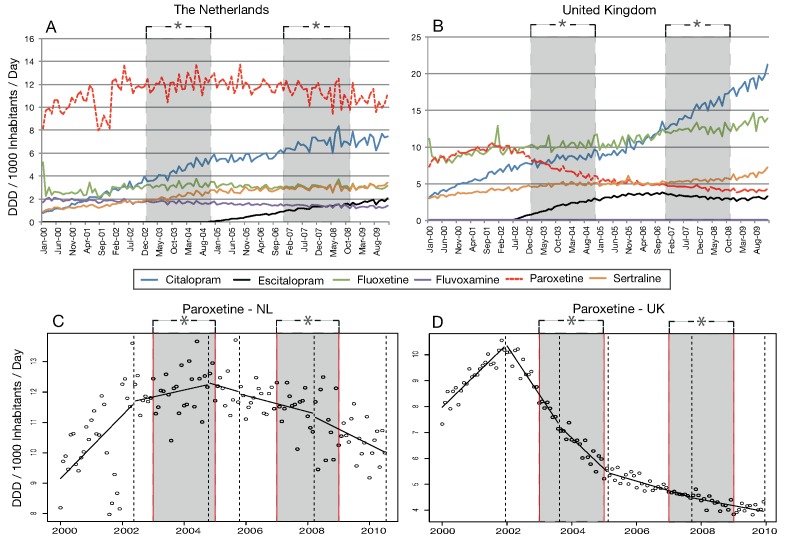
SSRI use in the NL (A) and in the UK (B), segmentation of paroxetine in the NL (C) and in the UK (D). Dotted lines represent a change in use trend and therefore a new, or the end of a segment. *The grey period illustrates the periods of media coverage of regulatory warnings.

When analyzing individual SSRI use in the NL, citalopram and escitalopram showed rapid growth (Figure 2A). Although the overall increase in paroxetine use was modest (8.2 to 10.0 DDD/1000/day), it remained the most frequently used SSRI in the NL. Regression analysis of paroxetine use demonstrated a rapid increase from January 2000 to May 2002, followed by a period of slowed growth until October 2004. At the end of the first period of media coverage of regulatory warnings, paroxetine use in the NL decreased consistently until July 2010 (Figure 2B, appendix table).

As in the NL, the use of citalopram and escitalopram increased exponentially in the UK in the period under survey. Fluoxetine, the most frequently used SSRI in the UK, demonstrated a modest increase of 11.2 to 13.9 DDD/1000/day during the period 2000–2010. Fluvoxamine use also demonstrated a consistent decrease during the entire study period in the UK, as was also documented in the NL. Overall paroxetine use decreased from 7.3 in January 2000 to 4.3 DDD/1000/day in December 2009 (Figure 2C, appendix table). Segmented regression analysis of paroxetine use revealed a rapid increase from January 2000 to January 2002, followed by a rapid decrease prior to the first period of media coverage of regulatory warnings. This downward trend persevered until December 2009 (Figure 2D).

### SSRI use in the NL Stratified by Specialty

**Figure 3:**
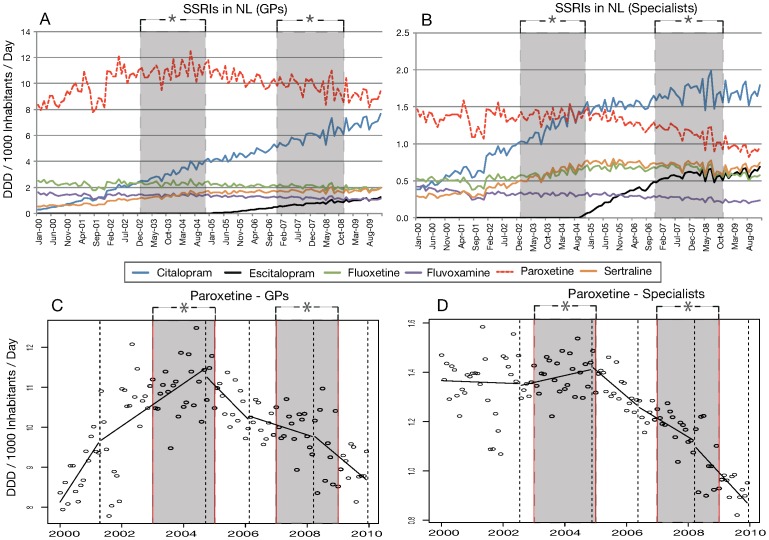
SSRI use in the NL through GPs (A) and specialists (B), segmentation of paroxetine use in the NL through GPs (C) and specialists (D). Dotted lines represent a change in use trend and therefore a new, or the end of a segment. *The grey period illustrates the periods of media coverage of regulatory warnings.

Dutch GPs prescribed the largest share of SSRIs (mean: 80.4%, 95% CI: 80.3; 80.6, Table 2). Therefore, national SSRI use trends and GPs’ SSRI prescribing trends were comparable (Figure 3A+B). Segmented regression analysis demonstrated that GPs steadily prescribed more SSRIs from January 2000 to September 2004 (appendix table). At the end of the first period of media coverage of regulatory warnings, SSRI prescriptions by GPs slightly decreased until January 2006 and then recovered to eventually reach a plateau from April 2008 to December 2009. Paroxetine GP prescriptions revealed an upward trend from January 2000 to September 2004 (appendix table). Towards the end of the first period of media coverage of regulatory warnings, GPs’ prescriptions for paroxetine showed a negative trend and continued decreasing until December 2009 (Figure 3C, appendix table). By December 2009, Dutch GPs’ citalopram prescriptions were almost level with paroxetine use (Figure 3A, appendix table). As far as paroxetine use is concerned, we see a downward trend in specialist prescriptions similar to the decrease in GPs’ prescriptions after the first period of media coverage in the NL (Figure 3D). The downward trend continued until December 2009 (Figure 3D, appendix table) and was molecule specific. Specialists’ prescriptions for Citalopram grew exponentially until the end of the first period of media coverage of regulatory warnings. Thereafter, growth slowed and following the second period of media coverage of regulatory warnings Citalopram use stabilized (appendix table).

**Table 2:** Number of DDD/1000 inhabitants/day of SSRIs in the Netherlands, and percentage between January 2000 and December 2009 stratified by medical specialty.

Specialty	General Practitioner				Specialists				Unidentified			
SSRI/Year	2000		2009		2000		2009		2000		2009	
Citalopram	0.3	(2.2)	7.6	(32.8)	0.4	(12.5)	1.8	(35.9)	0.02	(7.1)	0.2	(52.4)
Escitalopram*	0.002	(0.0)	1.2	(5.3)	0.01	(0.5)	0.7	(13.8)	0.001	(0.3)	0.02	(6.5)
Fluoxetine	2.5	(18.8)	2.0	(8.6)	0.5	(16.6)	0.6	(11.6)	0.06	(17.3)	0.0	(6.3)
Fluvoxamine	1.6	(12.1)	1.1	(4.9)	0.4	(14.0)	0.2	(4.7)	0.03	(10.0)	0.0	(4.4)
Paroxetine	8.4	(62.8)	9.4	(40.2)	1.5	(44.8)	1.0	(19.0)	0.20	(59.5)	0.1	(21.7)
Sertraline	0.5	(4.0)	1.9	(8.3)	0.3	(10.5)	0.7	(14.9)	0.01	(4.4)	0.0	(9.6)
**Total**	13.3	(100)	23.3	(100)	3.1	(100)	5.0	(100)	0.33	(100)	0.4	(100)
**Total (%) per specialty**	(79.5)		(81.1)		(18.5)		(17.4)		(2.0)		(1.5)	

* Data available from October 2004.

### SSRI use in the NL Stratified by Age Group

**Figure 4:**
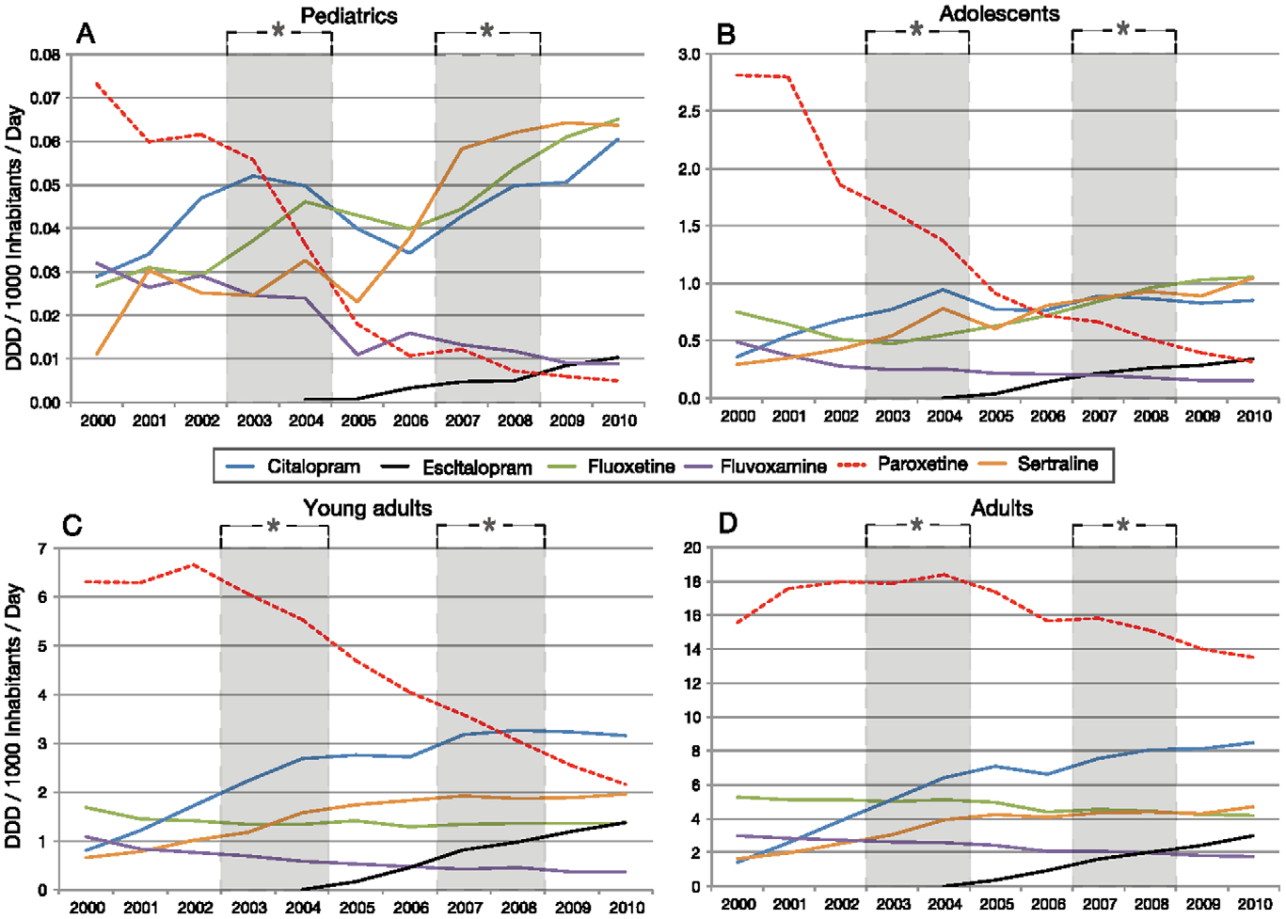
SSRI use in the NL in pediatrics (A), adolescents (B), young adults (C), and adults (D). *The grey period illustrates the period of media coverage of regulatory warnings.

In the NL, SSRI use in pediatrics, adolescents, and adults modestly decreased after the first period of media coverage of the warnings, and then recovered. Initially, the use of SSRIs increased in young adults; however, by the end of the first period of media coverage the use dropped until 2010. SSRI use by the elderly grew during the entire study period (data not presented). Specific Dutch SSRI trends revealed a growth in the use of citalopram, escitalopram, and sertraline across all age groups (Figure 4A–D). This growth was partially interrupted towards the end of the first period of media coverage of regulatory warnings, mainly in the younger groups (pediatrics, adolescents and young adults). The use of fluoxetine increased; however, only in pediatrics and adolescents. In adults and the elderly, the use of fluoxetine either remained stable or decreased modestly. A constant reduction in paroxetine use was measured prior to the first period of media coverage of regulatory warnings (2002) in pediatrics (from 0.06 to 0.005 DDDs/1000/day), adolescents (1.9 to 0.3 DDDs/1000/day), and young adults (6.7 to 2.2 DDDs/1000/day). Conversely, adults used more paroxetine in the period from 2000–2004 (15.5 to 18.4 DDDs/1000/day) than after the first period of media coverage of regulatory warnings when their use decreased to 13.5 DDDs/1000/day in 2010. A similar effect was measured in the elderly, as paroxetine use peaked in 2004 (14.5 DDDs/1000/day) and then decreased modestly after the first period of media coverage of regulatory warnings to 13.3 DDD/1000/day in 2010.

## Discussion

The regulatory authorities issued several warnings restricting the use of SSRIs less than 18 years of age between 2003–2004 due to uncertainties regarding the benefit/risk balance, and included further restrictions for young adults (18–24-years-old) in 2007–2008 [14]–[16],[19]. During these years, scientific journals and Dutch and British newspapers increased their (negative) coverage about the SSRI and suicidality controversy [21]. We analyzed British and Dutch SSRI use trends in 2000–2010 and assessed whether trend changes could be associated with the combined and long-term effect of both periods of media coverage of regulatory warnings. To our knowledge, this is the first study that presents such evidence on long-term use patterns of SSRIs and possible associations with media coverage of regulatory warnings. Trend changes in overall SSRI use largely corroborated with the periods of media coverage of the warnings. Both post-warning periods were associated with upward trends in SSRI use in the UK. Contrarily, Dutch post-warning periods were associated with limited reductions in overall SSRI use. However, these associations were not causal. In general, we found evidence of a temporal and limited association between overall SSRI use in both countries and both periods of media coverage of regulatory warnings. The effect of the periods of media coverage of regulatory warnings varied significantly per specific SSRI, country, and Dutch age groups. Stratified analyses showed a significant decrease in paroxetine use prior to the first period of media coverage of regulatory warnings in the UK overall and in Dutch pediatric, adolescent, and young adult age groups. Other SSRI use, especially (es)citalopram, continued to increase during the first period of media coverage of regulatory warnings in both the NL and UK. Still, paroxetine remained the most frequently used SSRI in the NL, whilst fluoxetine was used most frequently in UK in the 10-year period.

The present study has several strengths and limitations. The main strengths of this paper are the long-term analysis of trends of antidepressant use in the UK and the NL (based on national data), the comparison between two northern European countries, and the inclusion of all classes of antidepressants (not only those subject to safety advisories). Although media coverage represents only one of the many factors that may influence use (other factors might be reimbursement systems and policies, guidelines or patient compliance), our choice of the periods of media coverage of regulatory warnings is substantiated by a systematic analysis, which is also an important strength of the present study [21]. However, the present study also has limitations. Two distinct types of data on SSRI use were analyzed (IMS sales data for the NL and UK and Dutch GIP-prescription data). None of the datasets provided information on patient characteristics or detailed information on prescription dynamics at a patient level. Patient-level data can be used to assess trends in use over time on a more detailed level, such as rates of initiation of new prescriptions, discontinuation, or switching. However, these data were not available for the present study. We assessed a possible association between changes in Dutch and British antidepressant use and media or regulatory warnings on a national level, not on a micro level. Therefore, we used DDDs/1000/day to present drug utilization patterns. One of the greatest advantages of using the DDDs methodology when conducting drug utilization studies is that it enables comparisons between distinct molecules within and between countries. We consider that the quality of our data, the quantity, and interpretation in DDDs, were sufficient to answer our research question. However, further research in this direction could focus on analyzing antidepressant use and the influence of media and regulatory warnings at a patient level as mentioned above. Another weakness of our study is the lack of adjustment for pediatric doses. Unfortunately, the DDD-methodology is limited to adults, since the standard value assigned by the WHO is based on the main indication in adults. The lack of adjustment in our results creates an underestimation of the amount of antidepressant use in younger groups; this is unavoidable for drug utilization studies when analyzing pediatric off-label use. Unfortunately, due to the limited clinical evidence about the use of antidepressants in children, and the fact that dose calculations in children carry greater risks of error when compared with adults (differences in age and weight), no standardized guidelines for the use and dosage of antidepressants in children have been developed to date [29], [30]. Since we were interested in the macro-level dynamics/patterns of antidepressant use in children and the influence of media coverage of the warnings on use, we decided to present pediatric antidepressant use in DDDs despite all limitations. However, caution ought to be taken when interpreting the absolute level of use (number of DDDs/1000/day) in these young age groups.

The periods of media coverage of regulatory warnings had a limited and temporal effect on overall SSRI use in both the UK and NL. Significant reductions in SSRI use were not clearly observed during these periods. Overall SSRI use doubled during the period 2000–2010, which has been previously reported for other countries as well [6], [7], [10], [31]–[37]. It should be noted that overall antidepressant use could have increased significantly in the absence of regulatory actions or their coverage in the media, so the full effect of the regulatory actions or their coverage in the media may have been underestimated. When split by age groups, we observed that the increasing trend for Dutch SSRI use was temporarily interrupted in pediatrics, adolescents, and in less intensity in adults after the first period of media coverage of the warnings. Thereafter, SSRI use in these age groups recovered. Contrarily, SSRI use consistently decreased in young adults, whereas use by the elderly continued to increase despite media coverage of the warnings. These temporal decreases in SSRI use could indicate the prescribers’ attention and reaction to the warnings or media coverage. A similar response from prescribers to the regulatory advisories in children was reported for the UK, albeit without evidence of media influence [38].

Recent research on prescribing behaviors in the UK demonstrated that the increase in the prescriptions of antidepressants was not attributed to an increase of new patients (initiation), but to an increase in the number of long-term prescriptions [39]. Reasons for this growth in long-term use of antidepressants are to prevent relapses or recurrences, and to reduce the occurrence of withdrawal symptoms by titration and maintenance dosing. Research on antidepressant use in the NL in the 1990s demonstrated a similar cumulative effect in use, namely an increase in SSRI use both in terms of prevalence and incidence [40]. During the 2000s, the Dutch Health Insurance Board reported an increase in overall antidepressant use, while the number of SSRI users remained constant [41], demonstrating a shift in the 2000s when the prevalence of SSRI use increased, but the incidence did not. All in all, changes in the management of depression would be expected to affect population-level DDDs. Although this cumulative effect on antidepressant use was reported for both countries, UK national use was nearly two-fold higher than in the NL despite the use of DDDs as equivalent measure.

Towards the end of our study period in 2008, two important systematic reviews were published calling into question the effectiveness of SSRIs not only in pediatrics, but in adults and elderly, as well. In a meta-analysis, Kirsch et al. concluded that antidepressants were no better than placebo, and that in more severely depressed patients these drugs showed some effect, but only because of a poor response to placebo [42]. In the second publication, Turner et al. demonstrated that antidepressant trials with positive outcomes were published more often that those reporting negative outcomes [43]. This publication bias seemed to provide an incomplete picture when analyzing the efficacy of antidepressants by overestimating their efficacy. The publication of both systematic reviews, in particular Kirsch et al., evoked several media responses with controversial headers such as “depressing news, the happy pills don’t work”, or “anti-depressants taken by thousands of Brits ‘do NOT work’, major new study reveals” [44], [45]. Such publications, not related to the safety controversy, may also influence SSRI use. Despite this negative coverage in scientific journals and newspapers, SSRI use remarkably continued to grow in both countries after 2008. Overall SSRI growth in the UK was mainly driven by the use of citalopram, escitalopram, and fluoxetine. The UK guideline (NICE) for the treatment of depression recommends SSRIs, in particular (es)citalopram and fluoxetine, as first-line pharmacological interventions for the treatment of mild to severe depression based on their positive benefit/risk profile [46]–[48]. SSRIs growth could be attributed to these recommendations and the prescribers’ compliance. Another factor that could have influenced the increase in the use of escitalopram is its patented status (approved in 2002). However, this was not the case for citalopram that hitherto had shown a constant upward trend when its patent expired in 2003. Contrary to citalopram, paroxetine use dropped in February 2002, the same year that its patent status expired, and prior to the first period of increased (negative) media coverage and regulatory warnings. Most of the negative media coverage was directed towards paroxetine in both the NL and UK. In 2001, GlaxoSmithKline (GSK) lost its first lawsuit concerning paroxetine’s association with murder and suicide [49], [50], and this resulted in a FDA product warning [51]. In 2002, the BBC aired a documentary ‘The Secrets of Seroxat’ (paroxetine’s trademark) that highlighted safety concerns about this product, both in terms of suicidality and difficulties with discontinuing use [12]. These series of events may have induced the plunge in paroxetine use in the UK observed in our results prior to the first period of media coverage of regulatory warnings, in February 2002.

Specific SSRI use in the NL was comparable with the UK to a limited extent. Citalopram, escitalopram, and sertraline use also showed upward trends in the period under survey, albeit with limited signs of diminished use towards the end of the survey period and after the periods of media coverage of regulatory warnings. The Dutch GP guideline for the treatment of depression in adults recommends either a TCA or an SSRI as first-line treatment, giving priority to fluvoxamine, paroxetine, sertraline and a lower priority to fluoxetine due to the long-half life [52]. Remarkably, individual SSRIs with a large market uptake and a positive benefit/risk profile, such as citalopram and escitalopram [46]–[48] are not mentioned, nor recommended in the Dutch guidelines. The Dutch guideline for specialists extensively considers the benefits and risks of citalopram and escitalopram [53]. The preference for paroxetine in GP guidelines may be one of many factors why its use was less affected in the NL by media coverage of regulatory warnings compared to the UK [52] where citalopram, escitalopram, and fluoxetine are recommended for GP use. Most of the SSRI prescriptions in the NL were issued by a GP (±80%), confirming previous research [54]. Dutch GPs and specialists started prescribing less paroxetine towards the end of the first period of media coverage of regulatory warnings, apparently indicating a timely reaction from prescribers to the regulatory advisories or media attention. On the other hand, the increasing prescription rate of citalopram by both Dutch GPs and specialists demonstrated little or no effect during both periods of media coverage of regulatory warnings, as well as either prescribers’ disregard of the regulatory warnings or switching. The influence of guidelines, reimbursement policies, and prescribing habits for SSRI use should be further studied to better understand the differences for specific SSRIs and between countries.

Notwithstanding the modest reduction in paroxetine use in the NL, we measured significant drops in use for pediatrics, adolescents, and young adults prior to the period of media coverage of regulatory warnings. Therefore, no direct association between the periods of media coverage of regulatory warnings and decreased paroxetine use was found in young groups. Conversely, both periods of media coverage of regulatory warning were associated with decreased paroxetine use in adults and elderly, although the warnings (and updates) were originally not thought to affect these age groups. Presumably, disadvantages regarding the use of paroxetine, such as the high risk of withdrawal effects or akathisia, could have caused this reduction in use [55]. The first period of media coverage of regulatory warnings (2003–2004) was associated with a temporal dip in citalopram, and sertraline use in pediatrics, and adolescents in NL. Similar reductions in SSRI use by children and adolescents were also reported in other countries. [6], [7], [9], [56]–[58]. However, our data demonstrate that this temporal decrease in use by Dutch children and adolescent user groups recovered between the first and second period of media coverage of regulatory warnings. These results may indicate that doctors outweighed the benefits of SSRIs compared to the risks. Wijlaars et al. have reported similar long-term use patterns for British children, but without systematically accounting for the effects of the media coverage of the warnings, or differential antidepressant use by various young age groups [38].

### Conclusion

The timing of the media coverage of regulatory warnings about the suicidality risk associated with SSRI use coincided with changes in overall use in the NL and UK from 2000–2010. The results of this study demonstrate that short-term investigations only provide a snapshot of the potential implications of media coverage and regulatory warnings. We confirmed a strong, but not causal, association between periods of intense media coverage of regulatory warnings and significant changes in SSRI use over a ten-year period in both countries. However, our long-term assessment illustrated that the changes were temporal, drug-specific and more pronounced in pediatrics and young adults. The twofold increase in SSRI use over the 10-year period indicates that regulatory warnings and media coverage may come and go, but they do not have a significant impact on the overall upward trend of SSRI use as a drug class in both countries.

## Supporting Information

Box S1 **The SSRIs and suicidality controversy and regulatory decisions.**
(DOCX)

Appendix S1 **Segmentation of antidepressants in NL and the UK (TCAs, SSRIs, and other antidepressants).**
(EPS)

Appendix S2 **Segmentation of all SSRIs in the NL (paroxetine, sertraline, citalopram, escitalopram, fluoxetine, and fluvoxamine).**
(EPS)

Appendix S3 **Segmentation of all SSRIs in the UK (paroxetine, sertraline, citalopram, escitalopram, fluoxetine, and fluvoxamine).**
(EPS)

Appendix S4 **Segmentation of SSRI use in the NL through GPs (citalopram, escitalopram, fluoxetine, fluvoxamine, paroxetine, and sertraline).**
(EPS)

Appendix S5 **Segmentation of SSRI use in NL through specialists (SSRIs, citalopram, escitalopram, fluoxetine, fluvoxamine, paroxetine, and sertraline).**
(EPS)
