# Supplementary material for: A 10-Year Analysis of the Effects of Media Coverage of Regulatory Warnings on Antidepressant Use in The Netherlands and UK
Source: PLoS One. 2012 Sep 20;7(9):e45515. doi: 10.1371/journal.pone.0045515 (PMC3447767; doi:10.1371/journal.pone.0045515)
Supplement: Box S1 — The SSRIs and suicidality controversy and regulatory decisions. (DOCX) [file pone.0045515.s001.docx]

## Box S1

Box 1. The SSRIs and suicidality controversy and regulatory decisions

- In 2002, GlaxoSmithKline (GSK) submitted the results of pediatric clinical trials for the treatment of depression to the FDA requesting a 6-month market exclusivity for paroxetine (an SSRI) to treat childhood depression, as mandated by the Food and Drug Administration Modernization Act of 1997 (FDAMA) [[11](#_ENREF_11)].
- In 2002, negative media coverage claimed that GSK’s clinical data was altered to ‘limit negative commercial impact’ and data that revealed an increased suicide risk in children was not published [[12](#_ENREF_12),[13](#_ENREF_13)].
- In 2003-2004, worldwide regulatory agencies issued warnings banning the use of selective serotonin re-uptake inhibitors (SSRIs) in pediatrics (under 18-years-old), in reaction to safety concerns regarding the risk of suicidality [[14-16](#_ENREF_14)].
- In 2004, the Attorney General of New York State sued GSK for playing down the association between paroxetine use and suicidality in pediatrics by not publicizing negative clinical trial results. Paroxetine was not granted status as an indication for the treatment of childhood depression [[17](#_ENREF_17),[18](#_ENREF_18)].
- In 2007-2008, the previous warnings were revised resulting in the extension of the warnings to young adults (between 19 and 24 years of age) [[16](#_ENREF_16),[19](#_ENREF_19),[20](#_ENREF_20)].
